# A follow-up and treatment model for pediatric eating disorders: examination of the clinical variables of a child and adolescent psychiatry eating disorder outpatient clinic

**DOI:** 10.3389/fpsyt.2023.1218604

**Published:** 2023-09-28

**Authors:** N. Burcu Özbaran, Zeynep İrem Erbasan, Hazal Yağmur Yılancıoğlu, Didem Çek, Begüm Yuluğ Taş, Sibel Helin Tokmak, Tezan Bildik

**Affiliations:** ^1^Department of Child and Adolescent Psychiatry, Faculty of Medicine, Ege University, İzmir, Türkiye; ^2^Bakırçay University Çiğli Training and Research Hospital, İzmir, Türkiye

**Keywords:** anorexia nervosa, bulimia nervosa, eating disorder, clinical model, child and adolescence psychiatry

## Abstract

**Introduction:**

Anorexia nervosa and other eating disorders are common in children and adolescents and are characterized by symptoms such as food restriction, efforts to lose weight, fear of gaining weight and impaired body image. Anorexia nervosa is a life-threatening psychiatric disorder and its management in the outpatient setting can be challenging for clinicians. The aim of this study was to introduce the subunit service model developed for the multidisciplinary diagnosis and management of eating disorders in the outpatient setting and to evaluate the clinical follow-up of patients.

**Methods:**

The medical records of 37 patients who were followed up by the eating disorders team at our clinic between 2018 and 2022 were reviewed. The study was designed as retrospective case study.

**Results:**

A diagnosis was made according to DSM-5 and a treatment plan was developed for each case. Body mass index (BMI), Clinic Global Impression (CGI) scale scores, duration of follow-up, number of interviews and other scale scores (The Turgay Attention Deficit Hyperactivity Disorder Scale and the Autism Spectrum Screening Questionnaire Scale) of 37 patients aged 12-17 years diagnosed with an eating disorder and followed up in our clinic were statistically compared.

**Discussion:**

The Eating Disorder Follow-up Model developed and applied in our clinic had a positive effect on patients BMI scores, a significant improvement in CGI scores was observed. Conclusion: We believe that this multidisciplinary system will serve as a model for other mental health centers by raising awareness and guiding mental health professionals in the follow-up and treatment of eating disorders.

## Introduction

Eating disorders are psychiatric disorders that occur in childhood and especially in adolescence and can lead to significant consequences such as loss of function and mortality (1, 2). The prevalence of eating disorders has been reported to be 0.8% for anorexia nervosa (AN) and 0.28% for bulimia nervosa (BN) (3). Anorexia nervosa is an eating disorder (ED) characterized by symptoms such as restriction of food intake, efforts to lose weight, fear of gaining weight and impaired body image. Calorie counting, vomiting, use of laxatives and diuretics, and excessive exercise are common (4).

In the management of patients with eating disorders, a personalized treatment approach is recommended by providing patients with psychoeducation, symptom control, support with appropriate treatment methods and services for patients’ families (5). Managing a life-threatening condition such as AN in an outpatient setting can be challenging and hospitalization may be necessary, especially in severe cases. In clinical interviews, people with AN often deny psychopathology and adopt a negative attitude (6). The negative effects of food restriction on cognition make it difficult to establish therapeutic cooperation with patients. Difficulties in cognitive processes, low motivation for treatment and additional medical conditions in the early phase of treatment often require the involvement of the family in the process (7, 8). The course of the disease, often associated with organic complications, requires close contact with the pediatric unit. Nutritionists are consulted to provide appropriate nutritional support (9). Patients’ compliance and adherence to treatment may remain low despite support, and a resistant course of the disease may be observed. Therefore, the treatment of AN and other eating disorders should be individualized by a multidisciplinary team according to the patient’s needs. For all these reasons, clinicians should be able to manage a multidisciplinary process in the follow-up of patients with ED and, in particular, AN. The fact that the clinical manifestations of patients with AN are quite variable and that the causes of the disease are diverse shows that a good management approach is needed. In this regard, physicians should be provided with educational support (10).

In the treatment of eating disorders, it is known that inexperienced clinicians have difficulties in managing the therapeutic relationship and that treatment outcomes are negatively affected. It is thought that it is important to train experienced clinicians who can keep the therapeutic relationship strong in this disease with a high rate of resistance and dropout (11). There are studies in the literature that present therapy support and psychopharmacological approaches for AN (5). There are also studies sharing experiences in psychosomatic medicine and psychotherapy clinics (12). No studies have been found to improve eating disorder management skills in resident training. Considering that supporting child and adolescent psychiatrists’ ability to work with difficult cases will also increase the awareness of mental health professionals in the follow-up of eating disorders, this study was conducted to introduce and evaluate a sub-unit service model created for the multidisciplinary team and follow-up processes of eating disorder cases in outpatient settings at the Department of Child and Adolescent Psychiatry, Ege University. Before this model, patients and families complained of child and adolescent psychiatry residents rotating as part of their clinical duties, and they were often hesitant to re-explain their feelings to new rotationer residents, when their information was already available in patient files. In addition, the heavy workload in the university hospital created problems with information flow between residents.

The aim of the model developed is to establish a system in which the management of patients with eating disorders is carried out by a single clinician, a supervisor and a multidisciplinary team while at the same time facilitating the education and training of child and adolescent psychiatry residents in patient management and follow-up. In this sense, it is thought that this model could provide a dual benefit by simultaneously providing valuable patient care and maximizing training opportunities. In addition to the obvious benefits for patient care, the implementation of this model was deemed necessary for the training of all child and adolescent psychiatry residents for ED in our clinic. The aim of this study is to present the model for the treatment and follow-up of ED patients and to determine whether the model provides advantages in patient management compared with the rotation system.

## Materials and methods

The follow-up data of 37 patients who met the inclusion criteria among the patients followed up in our clinic between April 2018 and September 2022 as part of the Eating Disorder Follow-up “Ege Model” were presented in this study. Inclusion criteria were determined as clinically normal mental capacity, absence of neurological disease or chronic illness, and willingness to participate in the study. Exclusion criteria were determined as age under 12 years or over 18 years, unwillingness to participate in the study, clinically significant mental retardation, and concomitant neurological disease. The study was designed as a retrospective case study. Voluntary informed consent was obtained from all patients and their families.

### Ege eating disorders model summary

The Department of Child and Adolescent Psychiatry at Ege University provided services for children and adolescents aged 0–18 years and supervised the follow-up of patients in different sub-units according to age and diagnosis. Child and adolescent psychiatry residents, who received their specialist training in the department, rotated through all the sub-units of the clinic. In our clinic, ED cases were followed up by a team led by a supervisor (NBÖ) with experience in eating disorders and consultation-liaison psychiatry due to the frequent pediatric complications of ED. Due to the difficulty of following up ED patients in an outpatient setting, a consultancy system was developed to enhance the clinical management skills of child and adolescent psychiatry residents. This system allowed child and adolescent psychiatry residents to continue long-term management of their ED patient under consistent supervision even after they rotated to different sub-units. This also leads patients to be managed by a single clinician rather than by different child and adolescent psychiatry residents on new rotations. The team’s aim was to increase patient compliance and motivation and reduce drop-out rates through regular supervision.

In the follow-up of cases, the child and adolescent psychiatry resident conducted a comprehensive mental status assessment at the first meeting with the patient. The case was then presented to both the supervisor (NBÖ) and other child and adolescent psychiatry residents in the group receiving supervision. The patient’s follow-up was continued by the child and adolescent psychiatry resident who carried out the initial assessment, with the intervals between follow-ups being adjusted according to clinical need (daily, weekly or monthly) depending on the urgency of the case. A regular team meeting was held once a week, led by a supervising physician who had a medical service role in the university clinic as well as a teaching role. The supervisor was always available for emergencies due to the high incidence of comorbid mental illness, life-threatening complications and suicidal risk associated with AN. Emergency patients could be seen on a daily basis without waiting for the weekly meeting.

**Figure 1:**
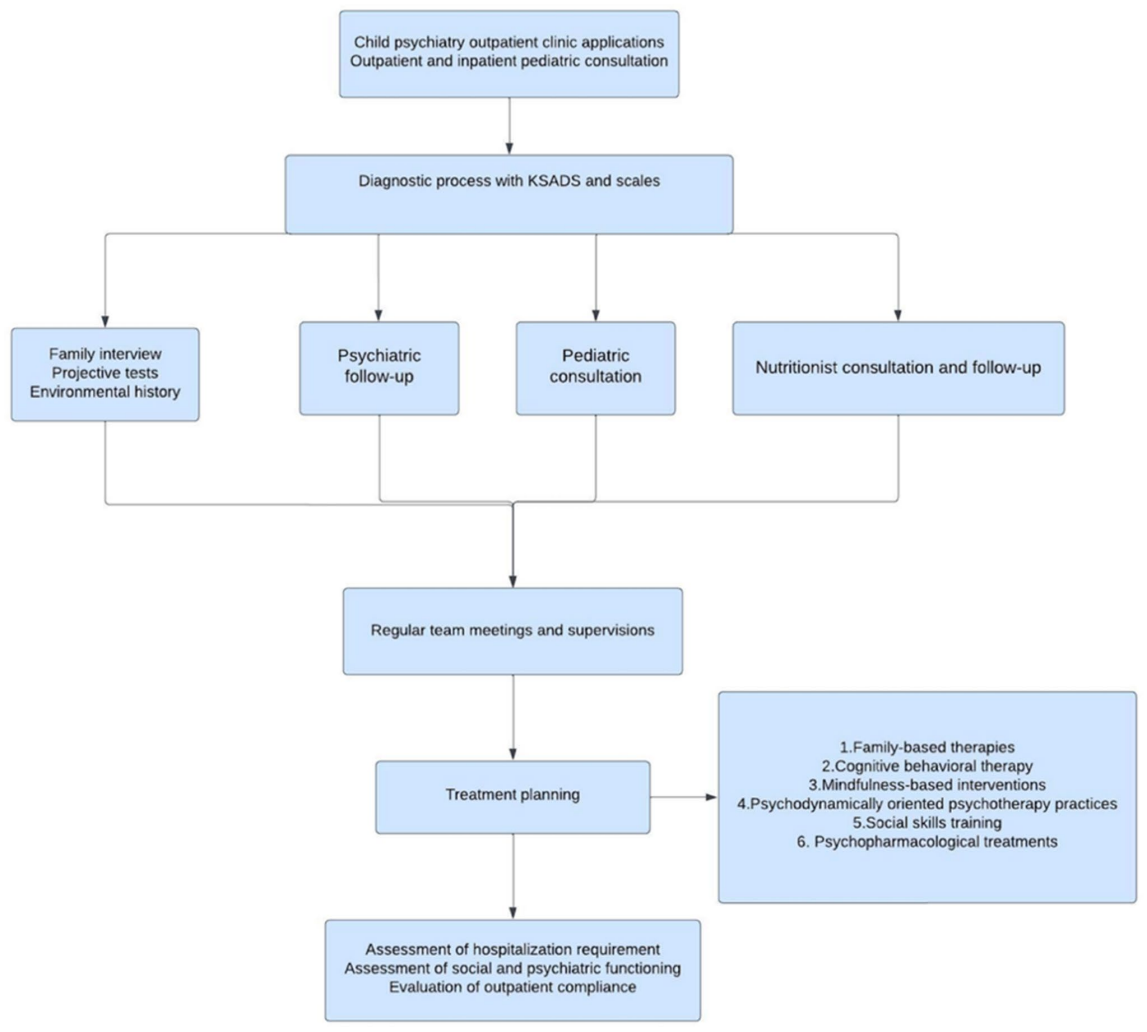
Eating disorder patient admission and follow-up flowchart.

The initial assessment of patients included an examination for clinical signs of eating disorders and a review of associated psychopathology (Figure 1). At this stage, motivational interviewing and cognitive behavioral therapy (CBT) techniques were used, depending on the clinical status of the patient. Disease severity was measured using the CGI in clinical interviews (13). Variables such as BMI, duration of clinical follow-up, number of visits, hospitalization status and medication use of the patients were included in the file data. The Turgay ADHD scale and the Autism Spectrum Screening Questionnaire (ASSQ) were routinely administered to each patient (14, 15). Additional assessments were made depending on the clinical status of the patient and additional scales were used. In addition, a comprehensive family history was taken for each case through family interviews conducted by the team psychologist. Consultation with adult psychiatry was sought if deemed necessary during the family interview. The environmental history was completed by collecting information from school forms. As part of the diagnostic and follow-up process, age-appropriate projective tests such as the Thematic Perception Test (16) and the Minnesota Multiphasic Personality Inventory (17) were routinely scheduled for each patient. A pediatric consultation was requested to screen for medical conditions. Cases were referred to the hospital dietitian for appropriate nutritional advice. The team also included a social worker. Following these comprehensive assessments, a Diagnostic and Statistical Manual of Mental Disorders-5 (DSM-5) diagnosis was made and a treatment plan was developed in collaboration with the supervising faculty member (NBÖ; Figure 1).

As long as the patient continues to cooperate, follow-up was continued until symptoms decreased and functionality reached a good level. The decision to discontinue was based on clinical opinion, patient request and supervision meetings. Due to the fact that an eating disorder can affect the medical condition and its consequences up to mortality, almost every patient was accepted as an emergency. There was no waiting list for assessment. Suicidality, psychotic symptoms, patients with poor compliance, patients whose medical status was compromised, rapidly progressing resistant cases were accepted as much more urgent cases. These patients were assessed for hospitalization and admitted to the pediatric and psychiatric wards as appropriate.

Within the framework of this model, the number of child and adolescent psychiatry residents monitoring eating disorder cases in our clinic during the study period was 43. The number of child and adolescent psychiatry residents exceeded the actual number of patients for the following reasons: All junior child and adolescent psychiatry residents joined the ED team when they started their residency and left when they finished. When child and adolescent psychiatry residents became specialists, they handed over their patients to other residents. In such cases, one patient was followed by two child and adolescent psychiatry residents.

### Ethical considerations

This study was conducted in accordance with the principles of the Declaration of Helsinki. Ethical approval was obtained from Ege University Medical Research Ethics Committee (decision number 22-6T/7). Voluntary informed consent was obtained from all participants and their families.

### Statistical analysis

Descriptive statistics were presented as mean, standard deviation, median, minimum, maximum, frequency and percentage. The normality assumption of quantitative data was tested using the Shapiro–Wilk test. The Mann–Whitney U test was used to compare non-normally distributed variables between groups. Relationships between quantitative variables were assessed using Spearman’s Rho correlation coefficient. Relationships between categorical variables were assessed using the Pearson chi-square test. Statistical analyses were performed using IBM SPSS Statistics 25.0 (IBM SPSS Statistics for Windows, Version 25.0. Armonk, NY: IBM Corp.). *p* < 0.05 was considered statistically significant in all analyses.

## Results

The follow-up data of 37 patients who met the inclusion criteria were presented in this study. The number of child and adolescent psychiatry residents involved in treatment and supervision was 43. A single physician provided supervision to all child and adolescent psychiatry residents. Team meetings were held on a weekly basis, with the exception of annual leave days during the summer months.

Sociodemographic data of the patients are presented in Table 1. Clinical characteristics such as eating disorder subtype, history of hospitalization, comorbidity, psychopharmacological treatment, follow-up period, and number of psychiatric interviews are presented in Table 2. Thirteen (35.1%) patients were reported to have symptoms of self-induced vomiting/using laxatives. It was observed that 10 (27.0%) patients experienced binge eating attacks. Twenty-six patients had ASSQ scores with a mean score of 7.19 ± 6.04. The difference between BMI measured at the last clinical interview and BMI measured at the first clinical interview was found to be significant (*t* = −7.378, *p* < 0.01). A negative correlation was found between BMI and ADHD-attention deficit scores at the first clinical interview (*r* = −0.495, *p* = 0.022). It was found that the BMI at the last clinical interview increased with increasing follow-up time (*r* = 0.561, *p* < 0.01) and number of interviews (*r* = 0.348, *p* = 0.038). The difference between CGI score in the last clinical interview and CGI score in the first clinical interview was found to be significant (*t* = 10.942, *p* < 0.01) (Table 3). It was observed that CGI scores in the last clinical interview decreased as the follow-up period of the patients increased (*r* = −0.364, *p* = 0.027). There were three (8%) patients who dropped out of follow-up. One patient (3%) dropped out due to moving to another city, and two patients (5%) dropped-out due to treatment incompliance.

**Table 1:** Sociodemographic characteristic of the cases.

	*N* (%)
**Gender**	
Female	35 (94.6)
Male	2 (5.4)
**Education**	
Middle school	9 (24.3)
High school	28 (75.7)
	**Mean ± SD**
Age	15.44 ± 1.61
Age of onset	14.46 ± 0.87
Time without treatment (month)	6.96 ± 6.66

SD, standard deviation.

**Table 2:** Clinical features of the cases.

	*N* (%)
**Eating disorder diagnosis**	
AN	34 (91.9)
BN	1 (2.7)
ED-NOS	2 (5.4)
**Hospitalization**	
Psychiatry	3 (8.1)
Pediatrics	9 (24.3)
Psychiatry + pediatrics	11 (29.7)
**Number of hospitalizations**	
None	14 (37.8)
Once	12 (32.4)
More than once	11 (29.7)
**Psychiatric comorbidity**	
Yes	28 (75.7)
No	9 (24.3)
Psychiatric comorbidity*	
MDD	19 (51.4)
Anx	9 (24.3)
ADHD	5 (13.5)
OCD	2 (5.4)
ASD	2 (5.4)
BD-NOS	2 (5.4)
**Medications**	
Yes	36 (97.3)
No	1 (2.7)
**Medications**	
SSRI	3 (8.1)
SSRI+AP	29 (78.3)
SSRI+AP + MTF	4 (10.8)
	**Mean ± SD**
Follow-up period (months)	11.81 ± 11.04
Number of interviews	19.11 ± 15.53

*Total percentage is above 100 since one patient can have more than one comorbid diagnosis. AN, anorexia nervosa; BN, bulimia nervosa; ED-NOS, eating disorder not otherwise specified; MDD, major depressive disorder; Anx, anxiety disorder; ADHD, attention deficit hyperactivity disorder; SSRI, selective serotonin reuptake ınhibitor; AP, antipsychotic; BD-NOS, bipolar disorder not otherwise specified; MTF, methylphenidate; SD, standard deviation.

**Table 3:** BMI and CGI values of the cases.

	Mean ± SD	*t*	*p*
**BMI**			
First clinical interview	15.48 ± 2.36	+7.378	<0.01*
Last clinical interview	20.01 ± 3.61		
**CGI**			
First clinical interview	5.27 ± 1.02	−10.942	<0.01*
Last clinical interview	2.95 ± 1.29		

BMI, body mass index; CGI, clinical global impression; SD, standard deviation; *t*, dependent *t*-test, *p < 0.05.

## Discussion

In this study, a treatment and follow-up model including child and adolescent psychiatry resident training in the field of eating disorders was presented and clinical data of the patients were evaluated. Before this model was developed, a system was used in which the core team (supervisor-dietician-psychologist-nurse) was the same and the child and adolescent psychiatry resident team rotated. In this old rotation system, the follow up period of cases by the child and adolescent psychiatry residents was short and the system failed to provide sufficient experience in patient management and follow-up. Although the rotation system could not be compared to the new model due to lack of data, a review of the literature has shown that inexperienced child and adolescent psychiatry residents, in particular, experience feelings of inadequacy, disappointment and hopelessness in relation to eating disorder patients and are reluctant to follow up eating disorder patients. This can disrupt therapeutic collaboration and affect treatment outcomes; therefore, it is critical to train well-qualified specialist physicians (11). One of the aims of this model was to increase the experience and competency of child and adolescent psychiatry residents with weekly team meetings, case presentations, sharing experiences, and regular supervision. The team worked on the basis of existing knowledge that a strong patient-doctor (child and adolescent psychiatry resident) relationship would reduce drop-out and increase treatment success (18).

Considering the various physical and psychiatric aspects of the disease, it has been strongly recommended that the management of ED should involve a multidisciplinary team consisting of psychiatrists, pediatricians, clinical psychologists, dieticians, nurses, social workers and occupational therapists (19). In our clinic, the management of ED cases was carried out in a multidisciplinary framework with a core team consisting of a consultant psychiatrist, a consultant child and adolescent psychiatrist, a pediatrician, a clinical psychologist, a dietician, a nurse and a social worker. Through all these disciplines, our clinic provided effective and comprehensive treatment for patients struggling with ED.

Guidelines with recommendations for the diagnosis, assessment, treatment, and management of eating disorders are available in the literature (20, 21). However, no study was found that described the clinical follow-up model for eating disorders.

The distribution of ED subtypes in the clinical profiles of the patients followed up with the model was determined as AN in 34 (91.9%) patients, bulimia nervosa (BN) in 1 (2.7%) patient and ED-NOS in 2 (5.4%) patients. In our study, 75.7% of patients had at least one comorbid mental disorder. Almost all patients received psychopharmacological treatment, and more than half were referred to inpatient wards (Table 2). These figures indicate that difficult patients were followed-up in our clinic. Nevertheless, significant improvements in CGI scores and BMI values were achieved (Table 2). The drop-out rate in our clinic was low compared to the literature (22). In this sense, the model is thought to have produced positive results in terms of therapeutic cooperation. In our model, treatment was individualized according to the specific needs of each patient. It included modalities that were consistent with those already recommended by guidelines, primarily consisting of CBT, supportive and dynamic therapies. Where necessary, psychotropic support was provided for ED or co-morbid conditions. Cases were referred to our inpatient ward in accordance with ED admission criteria. Patients with poor physical health were referred for pediatric hospitalization. Patients and families were interviewed at regular intervals. Supportive interviews focused on understanding the causality of the illness and the impact of outcomes. Anorexia nervosa is often seen in adolescence, and efforts are made to recognize the negative emotions and developmental challenges that characterize this phase. The treatment strategy aims to increase coping skills and develop interpersonal relationships. Treatment for weight gain is delivered parallel to social–emotional support (23).

### Strengths and limitations

Limitations of the study include factors such as the relatively small number of patients, the fact that the study was based on a model of patient follow-up, and the lack of a control group. The aim of the study was to present the model rather than demonstrate the effectiveness of the therapy. Therefore, the study focused on presenting the follow-up data of the patients. As all eating disorder patients in our clinic were followed with this model, there was no control group. Due to the lack of sufficient data on the rotation system used before this model, the differences before and after the model could not be measured. To the best of our knowledge, this is the first study to define and highlight the importance of an eating disorder-specific follow-up and treatment model. The results obtained show the usefulness of the model. The model used in our clinic does not claim to be a guideline in itself. However, it is presented as a method that can be applied in other intensive clinics that do not have a specific outpatient clinic for eating disorders. The number of eating disorder cases is increasing in today’s world and the training of child and adolescent psychiatry residents is becoming more difficult, this model can provide an example for future studies in other clinics focusing on eating disorders.

## Conclusion

The data of ED patients who were followed-up with regular meetings and counseling in collaboration with a fixed and established core team under a designated supervisor were presented in this study. The eating disorder follow-up model developed and applied in our clinic had a positive effect on patients’ BMI in correlation with follow-up time and number of interviews, and as a result, a significant improvement in CGI scores was observed. We believe that this multidisciplinary and continuous system will serve as a model for other mental health centers by raising awareness and guiding mental health professionals in the follow-up and treatment of eating disorders. The use of this and similar models will enable mental health professionals to make better clinical decisions and achieve better outcomes for patients with eating disorders in the future.

## Data availability statement

The raw data supporting the conclusions of this article will be made available by the authors, without undue reservation.

## Ethics statement

Ethical approval was obtained from Ege University Medical Research Ethics Committee (decision number 22-6T/7). Written informed consent was obtained from all participants and their families.

## Author contributions

NÖ: study design and method. ZE, BT, ST, DÇ, and HY: data collection. HY: analysis of data. NÖ, HY, ZE, and DÇ: preparation of original draft. NÖ and TB: review. All authors contributed to the article and approved the submitted version.
